# Uniform Cu-Based Metal–Organic Framework Micrometer Cubes with Synergistically Enhanced Photodynamic/Photothermal Properties for Rapid Eradication of Multidrug-Resistant Bacteria

**DOI:** 10.3390/pharmaceutics17081018

**Published:** 2025-08-06

**Authors:** Xiaomei Wang, Ting Zou, Weiqi Wang, Keqiang Xu, Handong Zhang

**Affiliations:** 1 Key Laboratory for Advanced Technology in Environmental Protection of Jiangsu Province, Yancheng Institute of Technology, Yancheng 224051, China; 224003011023@stu.ycit.edu.cn (X.W.); 234003011030@stu.ycit.edu.cn (T.Z.); 224003011068@stu.ycit.edu.cn (W.W.); 2 Department of Clinical Laboratory, Affiliated Hospital 6 of Nantong University, Yancheng Third People’s Hospital, Yancheng 224001, China

**Keywords:** photodynamic therapy, photothermal therapy, multidrug-resistant bacteria, metal-organic frameworks, antibacterial activity

## Abstract

**Background/Objectives**: The rapid emergence of multidrug-resistant bacterial infections demands innovative non-antibiotic therapeutic strategies. Dual-modal photoresponse therapy integrating photodynamic (PDT) and photothermal (PTT) effects offers a promising rapid antibacterial approach, yet designing single-material systems with synergistic enhancement remains challenging. This study aims to develop uniform Cu-based metal–organic framework micrometer cubes (Cu-BN) for efficient PDT/PTT synergy. **Methods**: Cu-BN cubes were synthesized via a one-step hydrothermal method using Cu(NO_3_)_2_ and 2-amino-p-benzoic acid. The material’s dual-mode responsiveness to visible light (420 nm) and near-infrared light (808 nm) was characterized through UV–Vis spectroscopy, photothermal profiling, and reactive oxygen species (ROS) generation assays. Antibacterial efficacy against multidrug-resistant *Escherichia coli* (*E. coli*) and *Staphylococcus aureus* (*S. aureus*) was quantified via colony counting under dual-light irradiation. **Results**: Under synergistic 420 + 808 nm irradiation for 15 min, Cu-BN (200 μg/mL) achieved rapid eradication of multidrug-resistant *E. coli* (99.94%) and *S. aureus* (99.83%). The material reached 58.6 °C under dual-light exposure, significantly exceeding single-light performance. Photodynamic analysis confirmed a 78.7% singlet oxygen (^1^O_2_) conversion rate. This enhancement stems from PTT-induced membrane permeabilization accelerating ROS diffusion, while PDT-generated ROS sensitized bacteria to thermal damage. **Conclusions**: This integrated design enables spatiotemporal PDT/PTT synergy within a single Cu-BN system, establishing a new paradigm for rapid-acting, broad-spectrum non-antibiotic antimicrobials. The work provides critical insights for developing light-responsive biomaterials against drug-resistant infections.

## 1. Introduction

Infectious bacterial diseases continue to pose a significant threat to global public health [1]. Currently, antibiotic therapy remains the most widely employed clinical strategy for combating bacterial infections. However, the extensive and often irregular application of antibiotics has directly contributed to the emergence and dissemination of antibiotic-resistant bacterial strains. A critical challenge is that the pace of novel antibiotic development significantly lags behind the rate of evolution and spread of resistant bacterial species, leading to the continuous emergence of multidrug-resistant bacteria, including so-called “superbugs”. Consequently, there is an urgent need to develop novel therapeutic strategies capable of effectively eliminating pathogens while circumventing existing antimicrobial resistance mechanisms to address the growing challenge of bacterial infections [2,3].

In the context of the development of non-antibiotic strategies, light-induced multimodal antibacterial therapy, including photodynamic therapy (PDT) [4,5] and photothermal therapy (PTT) [6,7], which have engendered renewed optimism for the field of antibacterial treatment, have emerged. In PDT, photosensitizers undergo photoactivation upon irradiation with specific wavelengths of light, generating cytotoxic reactive oxygen species (ROS), including superoxide radical (**·**O^2−^), singlet oxygen (^1^O_2_), and hydroxyl radical (**·**OH), which mediate the therapeutic effects [8]. ROS, such as ^1^O_2_ and **·**OH, can cause local chemical damage to proteins and DNA and induce bacterial apoptosis, thereby achieving effective sterilization without producing harmful by-products [9]. However, the short lifespan of ROS such as ^1^O_2_, which exhibits a half-life of approximately 48 ns in biological systems, limits their diffusion distance to ~20 nm within cellular environments [10]. In PTT, near-infrared (NIR) irradiation induces localized hyperthermia that mediates bactericidal effects through the structural disruption of cellular membranes. This temperature elevation increases membrane fluidity and permeability, ultimately causing bacterial death via thermal denaturation of critical proteins and irreversible biomolecular damage [11,12,13]. Although PDT and PTT have effective strategies in clinical applications, each therapy has some drawbacks. First, owing to the presence of hypoxia in the treatment area, meeting the oxygen requirements of PDT is difficult, which hinders ROS production [14]. For PTT, enhancing the photothermal conversion efficiency necessitates intricate modifications [15]. To achieve sufficient ROS production or temperature elevation, the laser power density is generally very high, which may damage healthy tissue [16].

In this study, we investigated the potential of PDT and PTT synergistic antibacterial therapy as an emerging antibacterial strategy, combining the advantages of both to achieve high efficiency, low toxicity, and a reduced risk of resistance development. The combination of light irradiation, which activates PDT and PTT, offers a promising approach to overcome the limitations of these individual therapeutic techniques [17]. On the one hand, the local increase in temperature caused by the photothermal effect can enhance the fluidity and permeability of the cellular membrane. Additionally, this process can facilitate the diffusion and reaction of ROS in bacterial cells, thereby strengthening the photodynamic bactericidal effect [18]. In contrast, the ROS generated during the photodynamic process can further damage bacterial cells, increasing their sensitivity to heat and thereby enhancing their photothermal bactericidal efficiency [19]. Additionally, photothermal and photodynamic processes can reinforce each other, forming a positive feedback loop that synergistically enhances antimicrobial efficacy. However, the design and synthesis of materials that combine PDT and PTT while maintaining biosafety are significant challenges.

Metal–organic frameworks (MOFs) are defined as porous materials with periodic network structures formed by the self-assembly of metal ions or metal clusters with organic ligands through coordination bonds [20]. MOFs possess a number of advantageous properties, including ultra-high specific surface areas, tunable pore structures and chemical compositions, excellent biocompatibility, and unique optical properties. These properties make them an ideal platform for photothermal and photodynamic synergistic antibacterial applications [21,22,23]. The combination of photothermal and photosensitive components, such as gold nanorods and black phosphorus quantum dots with photosensitive ligands such as porphyrins, is a typical approach to obtain MOFs with both PDT and PTT properties [24,25,26]. The implementation of these strategies necessitates the introduction of additional molecules to enhance PDT-PTT efficiency, which consequently results in complex synthesis steps and significant labor requirements.

While multicomponent composite-based approaches can potentiate synergistic therapeutic outcomes in PDT-PTT, synthetic complexity and operational challenges impede their translational potential. To address this issue, researchers have proposed an integrated strategy that simultaneously integrates photothermal and photodynamic functions in a single MOFs system. Han et al. synthesized a Cu MOF by doping Cu^2+^ into PCN-224, where Cu^2+^ captures electrons to inhibit recombination and enhance photothermal effects [27]. Li et al. reported that in Cu-TCPP MOFs nanosheets, TCPP ligands generate ROS under 660 nm laser irradiation, while d-d transitions at Cu^2+^ nodes enable efficient photothermal conversion [28].

However, the efficiency of light energy utilization in existing systems can still be improved. To broaden the light absorption of MOFs from the visible to the near-infrared (NIR) region, Nasi et al. thoroughly explored cation-exchange strategies for regulating MOF morphology, stacking patterns, and optical properties [29]. Notably, compared to MOFs based on Mn, Fe, Co, Ni, and Zn, Cu-MOFs exhibit superior visible and NIR light absorption, primarily due to their strong metal-to-ligand charge transfer (MLCT) character, which significantly boosts NIR absorption. Based on these insights, Cu^2+^ ions and organic ligands with extended π-conjugated systems (such as amino-or carboxyl-functionalized benzene rings) were selected. These ligands not only provide additional coordination sites but also modulate the MOF’s bandgap, narrowing it to enable efficient absorption across the visible and NIR regions, while further promoting MLCT and enhancing overall light-harvesting capability [30].

In this study, we developed a Cu-based MOFs (Cu-BDC-NH_2_, abbreviated as Cu-BN) with a broad-spectrum response and used a dual-light irradiation strategy (420 nm visible light + 808 nm NIR light) to maximize the synergistic effect between PDT and PTT. The Cu-BN micrometer cubic blocks synthesized via a one-step hydrothermal method showed an excellent photoresponse performance in both the visible and NIR regions. In the presence of dual-light irradiation, the material has dual functionality as a photosensitizer and can absorb visible light and produce ROS while simultaneously absorbing NIR light to achieve efficient photothermal conversion. The synergistic effect of PDT-PTT achieved extremely high antibacterial rates (99.96% and 99.86%, respectively) against multidrug-resistant *Staphylococcus aureus* (*S. aureus*) and *Escherichia coli* (*E. coli*) within 15 min of dual-light irradiation. Ultraviolet spectroscopic quantitative analysis confirmed that the conversion rate of ^1^O_2_ under dual-light irradiation reached 78.7%. These results indicate that cubic Cu-BN blocks have considerable potential as efficient and expeditious materials for combating drug-resistant bacterial infections.

## 2. Materials and Methods

### 2.1. Synthesis of the Cu-BN Micrometer Block

Cu-BN was synthesized using a one-step hydrothermal method [31]. Solution A: A quantity of 0.8 g of polyvinylpyrrolidone (PVP, Sinopharm Chemical Reagent Co., Ltd., Shanghai, China) was dissolved in 16 mL of a mixture of N,N-Dimethylformamide (DMF, Sinopharm Chemical Reagent Co., Ltd., Shanghai, China) and ethanol (volume ratio of 1:1). The solution was stirred for 5 min until a transparent solution was obtained. Solution B: Copper nitrate (Cu(NO_3_)_2_·3H_2_O, Sinopharm Chemical Reagent Co., Ltd., Shanghai, China) and 2-aminoterephthalic acid were dissolved in 16 mL of DMF, with continuous stirring until the solution changed from blue to blue-green. Solutions A and B were mixed and sonicated for 30 min. Next, the mixture was transferred to a Teflon-lined autoclave (Jinan Henghua Technology Co., Ltd., Jinan, China) and reacted at 100 °C for 8 h. The mixture was cooled to room temperature (25 °C). Then, the mixture was centrifuged at 3000 rpm for 1 min to collect the precipitate. The product was washed repeatedly until it was pure and bright green. Finally, the substance was vacuum-dried at 60 °C for 6 h to obtain the Cu-BN powder.

### 2.2. Characterization

X-ray diffraction (XRD) analysis was performed using a PANalytical X’Pert3 powder diffractometer (Malvern Panalytical, Almelo, The Netherlands), to assess crystalline phase composition. The morphology and microstructural features of the materials were evaluated via scanning electron microscopy (SEM) on a Nano SEM 450 system (FEI, Houston, TX, USA). The properties of Cu-BN dispersed in aqueous solution were further determined by dynamic light scattering (DLS, Malvern Zetasizer Nano ZS90, Malvern Panalytical, Worcester, the United Kingdom). Thermogravimetric (TG) and derivative thermogravimetry (DTG) analyses were conducted on NETZSCH STA449 C in N_2_ atmosphere with a heating rate of 10 °C min^−1^. Diffuse reflectance spectroscopy (200–900 nm) was carried out using a Shimadzu UV-2600 UV-visible spectrophotometer (Shimadzu, Kyoto, Japan), integrated with an integrating sphere attachment, employing barium sulfate (BaSO_4_, Sinopharm Chemical Reagent Co., Ltd., Shanghai, China) as the reference standard for reflectance calibration.

### 2.3. Photothermal Performance Test

The FLIR E5xt thermal imager (Teledyne FLIR, Wilsonville, OR, USA), was used to evaluate the photothermal performance. The experiments involved subjecting 200 µg/mL Cu-BN phosphate-buffered saline (PBS) to 420 nm visible light (Beijing Perfectlight PLS-SXE300^+^, equipped with a 420 nm bandwidth filter, 0.5 W·cm^−2^, Beijing Perfectlight, Beijing, China), 808 nm NIR light (Beijing BLUEPRINT laser, 1.0 W·cm^−2^, Beijing BLUEPRINT, Beijing, China), or a composite light source. The temperature changes were recorded at intervals of 1 min.

### 2.4. Antimicrobial Performance Test

Antibacterial activity was determined using the colony counting method. Multidrug-resistant *E. coli* (China Center of Industrial Culture Collection, No. 10663) and *S. aureus* (American Type Culture Collection, No. 43300) were selected as model strains. These strains were inoculated into Luria-Bertani (LB, Bkmam, Changde, China) liquid medium and cultured in a shaking incubator, Yiheng, Shanghai, China) at 37 °C until the late logarithmic growth phase. The bacterial suspension was then diluted with phosphate-buffered saline (PBS, Bkmam, Changde, China) to a concentration of 1 × 10^5^ CFU/mL. A mixture of 180 μL of the diluted bacterial solution and 20 μL of the test material suspension (0.5 mg/mL, 1.0 mg/mL, and 2.0 mg/mL) was added to a 96-well plate (Bkmam, Changde, China). and exposed to different light sources (420 nm visible light, 808 nm near-infrared light, and 420 nm + 808 nm dual light) for 15 min. After light exposure, the bacterial suspensions from each well were collected and mixed, and 100 μL of the mixture was evenly spread onto the surface of LB solid medium (Bkmam, Changde, China), using sterile glass beads (Bkmam, Changde, China). After incubating at 37 °C for 48 h, photographs of the colonies on the plates were taken, and the number of colonies was counted using ImageJ software (V1.53t). The formula for calculating antimicrobial efficiency is as follows.

Antibacterial efficiency (%) = (Nc − Ne)/Nc × 100%, Where Nc is designated as the number of colonies in the control group, and Ne signifies the corresponding number of colonies in the experimental group.

### 2.5. Biocompatibility Assay

The CCK-8 assay was employed to measure the relative cell viability of human esophageal squamous cell carcinoma (KYSE-510, Procell, Wuhan, China) cells after co-culturing with the supernatants of Cu-BN at different concentrations (25, 50, 100, 200 μg/mL) for 12 h at 37 °C.

### 2.6. Detection of Singlet Oxygen Release

Add 5 mg of the test material to 1 mL of a 5 mM 1,3-diphenylisobenzofuran (DPBF Adamas, Shanghai, China) solution in dimethyl sulfoxide (DMSO, Adamas, Shanghai, China) and disperse evenly. Expose the dispersion to irradiation under a xenon lamp equipped with a 420 nm bandwidth filter (Beijing Perfectlight, Beijing, China), 808 nm near-infrared light (Beijing BLUEPRINT, Beijing, China) and dual-light composite for 15 min to assess ^1^O_2_ production. Record the UV-Vis spectrum of DPBF and its absorbance at 416 nm. The formula for calculating the release rate of ^1^O_2_ is as follows:

^1^O_2_ conv. (%) = (Ab_0_ − Ab_e_)/Ab_0_ × 100%, Where Ab_0_ denotes the relative intensity of ultraviolet absorption at 416 nm for 5 mM DPBF, and Ab_e_ denotes the relative intensity of ultraviolet absorption at 416 nm for the experimental group.

## 3. Results and Discussion

**Scheme 1:**
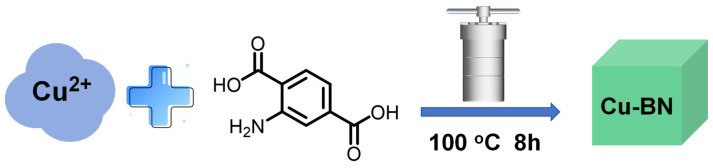
Schematic illustration of the preparation of Cu-BN.

The fabrication process of Cu-BN is illustrated in Scheme 1. Cu-BN was synthesized using the sol-gel method with Cu(NO_3_)_2_ as the metal source and 2-amino-p-benzoic acid (H_2_BDC-NH_2_) as the ligand. Under the promotion of the protective agent PVP, the coordination between Cu^2+^ and H_2_BDC-NH_2_ promoted the formation of uniform Cu-BN microparticles in ethanol and DMF.

**Figure 1:**
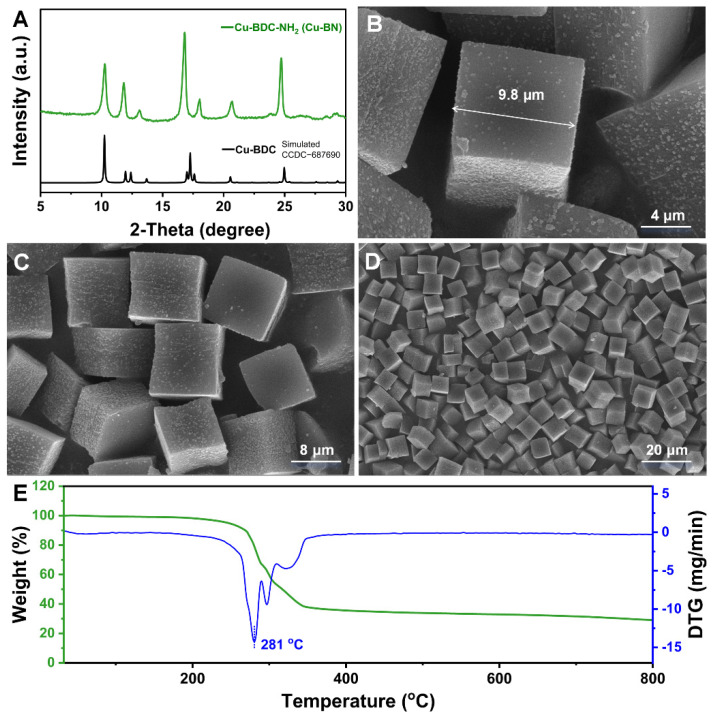
(**A**) XRD pattern, (**B**–**D**) SEM images, and (**E**) TG-DTG curve of Cu-BN.

As illustrated in Figure 1A, the principal XRD diffraction peaks of Cu-BN are predominantly located within 2θ = 5–30°. The intensity of these diffraction peaks indicates a well-ordered crystalline structure, confirming the successful synthesis of a material with high crystallinity. However, due to the unavailability of single-crystal X-ray diffraction (SCXRD) data for Cu-BDC-NH_2_, we compared its XRD pattern with that simulated from the SCXRD data of the structurally analogous Cu-BDC (CCDC No. 687690). Notably, the XRD pattern of Cu-BN exhibits minor deviations in peak positions and some peaks absent relative to those of Cu-BDC. These variations can be attributed to slight structural modifications induced by –NH_2_ functionalization of the ligand [32]. Given the lack of SCXRD data, it is not possible to definitively confirm complete structural similarity between Cu-BDC-NH_2_ and Cu-BDC. Consequently, we acknowledge the current limitations in fully verifying the crystal structure of Cu-BN. This aspect is presented as an open question for further investigation by the scientific community, potentially through the application of advanced microcrystal diffraction techniques such as micro-focused synchrotron XRD or electron diffraction. The microstructure of the Cu-BN nanomaterial was observed via SEM (Figure 1B–D). The results showed that the material had a highly uniform cubic structure with a side length of about 9.8 μm, featuring low surface roughness and distinct edge contours. The DLS test results indicate that the polydispersity index (PDI) value of the Cu-BN micromodules is 0.288, and the zeta potential is −7.73 mV. The thermal stability of Cu-BN was further confirmed by thermogravimetric analysis (TGA). As shown in Figure 1E, the TG curve reveals that Cu-BN exhibits negligible weight loss up to 200 °C, indicating excellent thermal stability under heating conditions. The onset decomposition temperature is observed at approximately 281 °C, demonstrating excellent thermal stability suitable for photothermal applications requiring prolonged irradiation.

**Figure 2:**
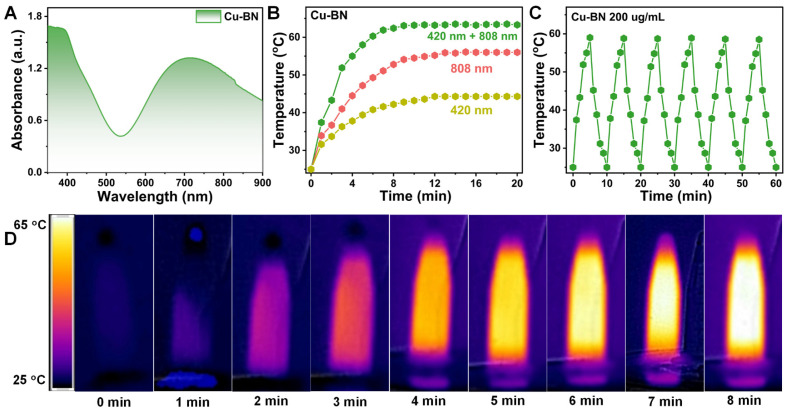
(**A**) Solid-state UV–Vis absorption spectra of Cu-BN. (**B**) Photothermal curves of Cu-BN after exposure to different light sources for 20 min. (**C**) Cyclic photothermal stability of Cu-BN under repeated dual-light on/off cycles. (**D**) Corresponding real-time infrared thermal images of Cu-BN.

The results of the UV–Vis diffuse reflectance spectroscopy (UV–Vis DRS) analysis (Figure 2A) revealed that Cu-BN has broad-spectrum absorption characteristics, demonstrating excellent light absorption performance in the visible and NIR regions. This phenomenon is attributed to the bandgap-matching effect of the material, which improves its ability to capture visible light and its photothermal conversion potential. Under single-wavelength irradiation (420 nm or 808 nm), the temperature increase rate of the Cu-BN was constrained (Figure 2B). However, under dual-light synergistic irradiation (420 + 808 nm) for 5 min, the material temperature rapidly increased from 25 °C to 58.6 °C. This increase was significantly greater than the one obtained through irradiation by a single light source. This finding indicates that visible–NIR dual-light excitation can trigger a synergistic photothermal enhancement mechanism. Further validation of the photothermal stability of the material was conducted via cyclic heating and cooling experiments (Figure 2C). Cu-BN showed consistent thermal stability during six consecutive cycles of dual-light irradiation (5 min) followed by cooling (natural cooling to room temperature), with the peak temperature maintained within the range of 60 ± 1 °C. No significant decay was recorded in the heating rate, which indicated the excellent thermal stability of Cu-BN.

**Figure 3:**
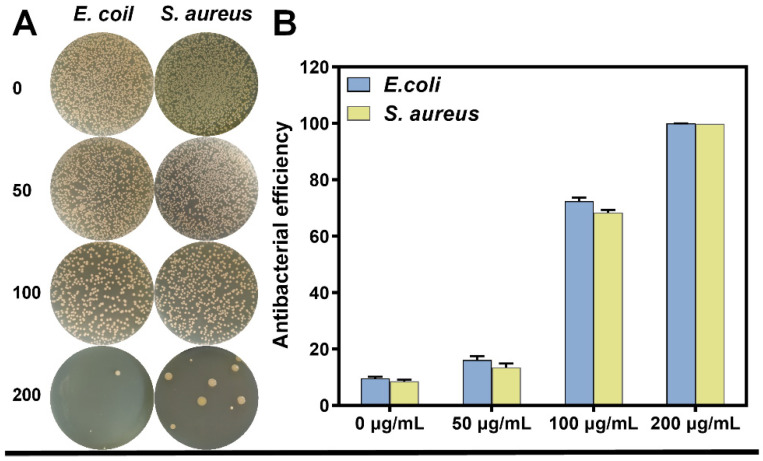
(**A**) Images of bacterial colonies generated from multidrug-resistant *E. coli* and *S. aureus* and (**B**) the corresponding antibacterial potency at different concentrations ranging from 0 to 200 µg/mL after exposure to dual light for 15 min.

The antibacterial efficacy of the material against *E. coli* and *S. aureus* was evaluated by the colony counting method. The antibacterial efficiency of the Cu-BN dispersions at various concentrations (50, 100, and 200 μg/mL) under composite light irradiation at 420 nm and 808 nm against the two bacteria is shown in Figure 3. In the context of dual-light irradiation at 420 and 808 nm for 15 min, the presence of Cu-BN resulted in a substantial synergistic PDT-PTT enhancement effect. Using the photothermal effect of the [Cu_2_(COO)_4_] metal cluster at 100 μg/mL, Cu-BN showed a strong bactericidal effect against *E. coli* and *S. aureus*, with rates of 72.4% and 68.4%, respectively. However, at 200 μg/mL, the PDT-PTT synergistic mechanism was found to overcome the structural barrier of Gram-positive bacteria, achieving bactericidal rates of 99.94% and 99.83% against *E. coli* and *S. aureus*, respectively. This concentration-dependent synergistic effect confirms the ability of Cu-BN to overcome bacterial structural resistance, thus establishing the basis for further investigation into its photoresponsive mechanisms.

**Figure 4:**
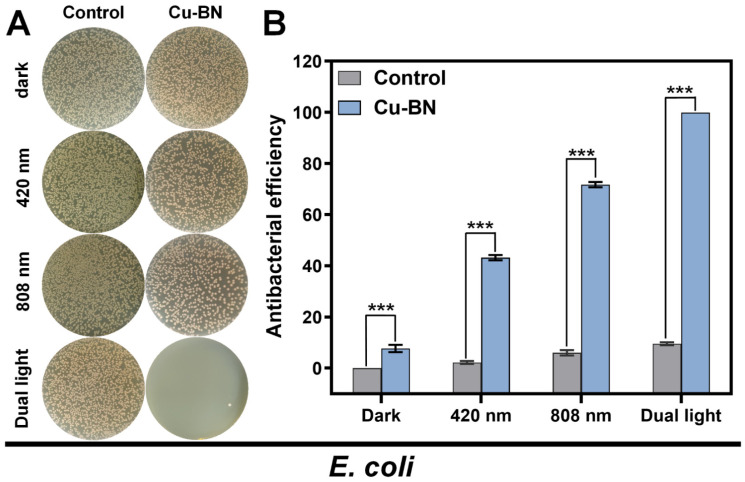
(**A**) Images of bacterial colonies generated from multidrug-resistant *E. coli* and (**B**) the corresponding antibacterial potency under dark conditions or upon exposure to irradiation with 420 nm light, 808 nm NIR, and dual light for 15 min. (n = 3, *** *p* < 0.001).

To investigate the effects of light-induced modes on the antibacterial properties of materials, a systematic analysis was conducted on the bactericidal efficacy of Cu-BN (200 μg/mL) against *E. coli* under different light irradiation conditions (420 nm, 808 nm, and dual-light synergistic irradiation) (Figure 4). Control experiments showed that single light irradiation (420 nm, 808 nm, or dual light) did not result in significant antibacterial activity (bactericidal rate < 10%), confirming that the light-induced antibacterial effect depends fully on the photoresponsive properties of the material. In the context of single-light irradiation at 420 nm, Cu-BN exhibited a bactericidal rate of 43.2%, which was attributable to the occurrence of visible light-driven PDT. In contrast, under 808 nm single-light irradiation, the bactericidal rate increased to 71.7%, a phenomenon attributable to the PTT effect. This finding suggests that PTT has a superior penetration advantage under a single antibacterial mode. This phenomenon can be attributed to the short lifetime (<40 ns) and limited diffusion distance (~20 nm) of ROS, which hinders their penetration into bacterial biofilms. In contrast, the photothermal effect disrupts the bacterial membrane lipid bilayer through sustained thermal effects, leading to intracellular protein denaturation and inactivation. The dual-light synergistic irradiation can significantly enhance antimicrobial efficiency; the bactericidal rate of Cu-BN increases to 99.94%. Under the dual-light synergistic effect, the bactericidal rate is increased to a level that nearly achieves complete sterilization through the PDT-PTT synergistic mechanism. The results presented here confirmed the spatiotemporal complementarity of dual-light synergism between PDT (ROS generation) and PTT (thermal effect), thereby validating the efficient elimination of drug-resistant bacteria.

**Figure 5:**
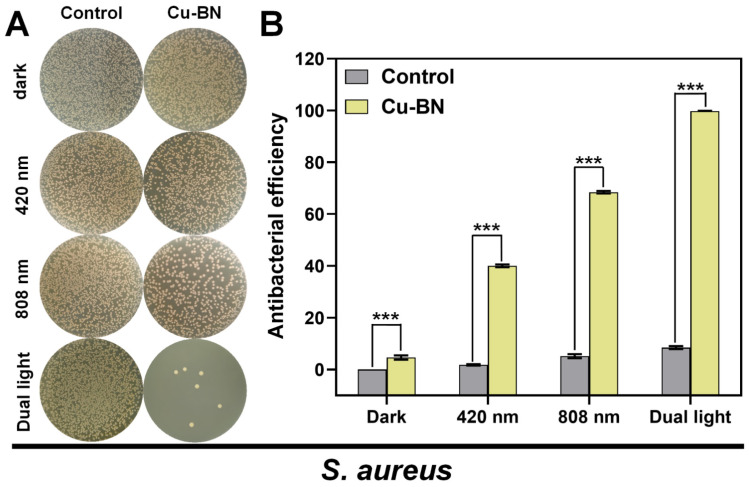
(**A**) Images of bacterial colonies generated from multidrug-resistant *S. aureus* and (**B**) the corresponding antibacterial potency of multidrug-resistant *S. aureus* under dark conditions or after exposure to irradiation with 420 nm light, 808 nm NIR, and dual light for 15 min. (n = 3, *** *p* < 0.001).

The regulatory mechanism underlying *S. aureus* antimicrobial performance was evaluated using the plate count method to assess the bactericidal efficacy of Cu-BN under different light conditions (Figure 5). In the context of monochromatic light irradiation at 420 nm, Cu-BN showed a bactericidal rate of 40.0%, which was attributable to the PDT effect. However, under 808 nm monochromatic light irradiation, the bactericidal rate increased to 68.5%, which was a result of the PTT effect. This PTT effect was less pronounced in *E. coli*, with a recorded bactericidal rate of 71.7%. This finding reinforces the notion that the photothermal effect results in greater penetration of Gram-positive thick-walled bacteria. In the context of dual-light synergistic effects, the bactericidal rate of Cu-BN increased to 99.83%, and Cu-BN also showed a significant photomodulatory synergistic enhancement effect on *S. aureus*. This outcome confirmed the universality of the PDT-PTT synergistic mechanism across different bacterial strains, thereby highlighting the broad-spectrum antibacterial potential inherent in the material design.

**Figure 6:**
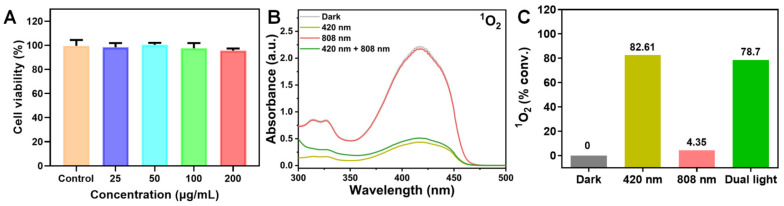
(**A**) CCK-8 assay of the relative cell viability of KYSE-510 cells co-cultured at diverse concentrations of the Cu-BN for 12 h. (**B**) Liquid UV–Vis spectra of ^1^O_2_ conversion under different light sources after 15 min of irradiation. (**C**) ^1^O_2_ conversion rates of Cu-BN under different light sources after 15 min of irradiation.

The biocompatibility of Cu-BN is a crucial factor limiting its biological applications. Using human esophageal squamous cell carcinoma (KYSE-510) as a cellular model, the cytotoxicity of the Cu-BN was evaluated through the CCK-8 assay. After co-culturing the cells with the supernatants of Cu-BN at different concentrations (25, 50, 100, 200 μg/mL) for 12 h, they exhibited remarkable relative viability. Remarkably, even at a concentration of 200 μg/mL, the viability of KYSE-510 cells remained above 95.65% (Figure 6A). The results powerfully demonstrate that the Cu-BN has no substantial cytotoxicity or adverse effects on the survival of human cells.

The generation characteristics of ^1^O_2_ in Cu-BN under visible light at 420 nm, NIR light at 808 nm, and dual-light excitation were systematically analyzed via ultraviolet spectroscopy [33]. As illustrated in Figure 6B, DPBF was used as a probe, and the relative generation rate of ^1^O_2_ was evaluated by monitoring the extent of decay of its characteristic absorption peak (416 nm). Cu-BN showed extremely low ^1^O_2_ conversion rates under both dark conditions and 808 nm NIR alone (Figure 6C), with negligible changes observed over 15 min. This finding suggests that Cu-BN has a low ^1^O_2_ generation efficiency under conditions of no light or NIR irradiation alone. However, under 420 nm visible light irradiation, the conversion rate increases to 82.61%. The conversion rate decreased to 78.7% under dual-light composite irradiation. This phenomenon can be attributed to several factors, including the brief duration of ^1^O_2_ generation, which was associated with a rapid increase in temperature under dual-light irradiation. This acceleration in temperature accelerated the escape of ^1^O_2_, consequently reducing the observed ^1^O_2_ conversion rate. This phenomenon elucidates the dynamic equilibrium between photothermal effects and ROS generation, thereby providing experimental evidence for the optimization of light energy allocation strategies.

## 4. Conclusions

To summarize, we used an integrated design strategy to construct Cu-BN micron-sized blocks with dual-mode responses to visible light and NIR light, thereby achieving the synergistic integration of PDT and PTT within a single material system. The experimental results showed that when the material was exposed to a combination of 420 nm visible light and 808 nm NIR light, it efficiently eliminated *E. coli* (99.94%) and *S. aureus* (99.83%) within a time frame of 15 min. This significant antibacterial activity is attributable to the synergistic mechanism of PDT-PTT, whereby photothermal effects increase membrane permeability by disrupting the bacterial membrane lipid bilayer while concomitantly promoting the diffusion of ROS. Simultaneously, ^1^O_2_ generation by PDT causes oxidative damage to bacterial components and enhances their thermal sensitivity. This study provided a theoretical basis and design framework for developing novel antimicrobial materials with broad-spectrum antibacterial activity, rapid responsiveness, and structural stability. These materials have applications in clinical antimicrobial therapy, smart wound dressing development, and biomaterial.

## Data Availability

The original contributions presented in this study are included in the article. Further inquiries can be directed to the corresponding authors.
